# Alleviative Effect of Probiotic Ferment on *Lawsonia intracellularis* Infection in Piglets

**DOI:** 10.3390/biology12060879

**Published:** 2023-06-17

**Authors:** Tingting Xu, Yong Guo, Yuanyuan Zhang, Kai Cao, Xinchen Zhou, Mengqi Qian, Xinyan Han

**Affiliations:** 1 Hainan Institute, Zhejiang University, Yazhou Bay Science and Technology City, Sanya 572025, China; 22017016@zju.edu.cn (T.X.); 22117076@zju.edu.cn (X.Z.); 2 Key Laboratory of Animal Nutrition and Feed Science in East China, Ministry of Agriculture, College of Animal Sciences, Zhejiang University, Hangzhou 310058, China; 0017668@zju.edu.cn (Y.Z.); 22117012@zju.edu.cn (K.C.); 22017017@zju.edu.cn (M.Q.); 3 Institute of Animal Husbandry and Veterinary Science, Zhejiang Academy of Agricultural Sciences, College of Animal Science and Technology, Hangzhou 310022, China; 13858038680@163.com

**Keywords:** *Lawsonia intracellularis*, probiotic ferment, intestinal function, porcine ileitis

## Abstract

**Simple Summary:**

*Lawsonia intracellularis (LI)* is a pathogenic bacterium in pig ileitis, which will cause the thickening of ileal mucosa and slow and uneven growth in pigs. Currently, the global positive rate of colitis is gradually increasing. It is estimated that the annual loss per infected pig exceeds 3–11 dollars, seriously increasing the cost of breeding production. Due to the high cost of commercial vaccines and immune function acquired three weeks later, there are fewer vaccines used in actual production. This experiment investigated the effects of probiotic ferment added to the diet on the growth performance, digestion, absorption, and intestinal function of piglets infected with *LI*, to explore the efficiency and mechanism of probiotic ferment in alleviating *LI* infection in piglets. The results suggest that probiotic ferment can reduce the colonization of *LI* in the ileum, improve intestinal damage, barrier function and microbiota structure, and enhance digestive enzyme activity and nutrient transport proteins expression, thereby improving piglet growth performance, which has the effect of preventing ileitis in pigs, to provide a scientific basis for the prevention of pig ileitis and its application in the aquaculture industry.

**Abstract:**

(1) Background: *Lawsonia intracellularis* (*LI*) is an obligate intracellular Gram-negative bacterium that causes porcine ileitis. Pigs infected with *LI* have severe ileal lesions and show symptoms of diarrhea, indigestion, and growth retardation. Previous studies found that probiotic ferment (FAM) improved the growth performance, gut barrier, and function in piglets. Therefore, we aimed to reveal the mechanism that FAM alleviates negative performance in *LI*-challenged piglets by characterizing the changes in intestinal integrity, function, and gut microbiota following FAM supplementation. (2) Methods: Twenty-four healthy piglets were randomly allotted to four treatments. Three groups were challenged with *LI*; both FAM addition and vaccination were performed to explore their positive effects on *LI*-infected piglets. (3) Results: Piglets infected with *LI* showed lower growth performance and typical pathological symptoms. Moreover, microscopic images showed that observed intestinal morphological damage could be repaired by FAM and vaccine. To explore the digestion of nutrients in piglets, both digestive enzyme activity and ileal transporter expression were performed to reveal the promoting effect of additives. Reduction of *LI* colonization intervention by FAM could also ameliorate abnormal differentiation and function of intestinal epithelial cells and alleviate severe inflammatory responses in piglets. Regarding the gut microbiota, both the structure and function of the ileal and colonic microbiota were altered following FAM supplementation. (4) Conclusions: In conclusion, probiotic ferment can reduce the colonization of *LI* in the ileum, improve intestinal damage, barrier function and microbiota structure, and enhance digestive enzyme activity and nutrient transport proteins expression, thereby improving piglet growth performance, which has the effect of preventing ileitis in pigs.

## 1. Introduction

Lawsonia intracellularis (*LI*) is an obligate intracellular Gram-negative bacterium classified as *Desulfovibrio* that causes porcine proliferative enteropathy in many animals, especially pigs [1]. Since the lesions of infection are concentrated in the ileum and can partially extend to the jejunum or colon, it is also called porcine ileitis (PI) [2]. Generally, *LI* increases infection with other enteric pathogens and leads to a variety of clinical symptoms, which includes anorexia, diarrhea, growth retardation, and even death due to severe intestinal bleeding [3,4]. The ileum also shows different degrees of intestinal enlargement, mucosal thickening, mesenteric congestion, or other pathological symptoms. Additionally, *LI* in the excreta of infected pigs can survive up to two weeks in moderate-temperature environments and spread rapidly in the herd through the oral–fecal route [2,5]. Therefore, PI has seriously affected the health and welfare of pigs and caused economic losses worldwide [6].

*LI* achieves rapid movement outside the host cells by means of monopolar flagella, which may also be a key factor in its penetration through the mucosal layer and into the intestinal epithelium [7,8]. While it has been demonstrated that bacteria will internalize into cells by forming vesicles rich in *LI* [9], infected cells transmit bacteria to daughter cells through mitosis or are released from the cytoplasm to infect other susceptible cells, leading to abnormal growth and inability to mature of crypt cells, gradually forming a clinical phenomenon of crypt proliferation [10]. As a result, the immature intestinal crypt epithelial cells proliferated and could not differentiate into functional cells, which damaged the intestinal mucosal barrier function significantly and further aggravated the infection [11,12].

At present, a commercial vaccine has been used to prevent porcine ileitis through drinking water or oral administration. While antibiotics need to be avoided before and within three days after vaccination, after vaccination, there is usually a lag period of three to four weeks, which leads to the development of protective immunity. Therefore, early immunization of piglets is necessary [13]. Oral or intraperitoneal injection of attenuated live vaccines can produce antibody responses similar to natural infections but with lower intensity and speed than natural infections [14]. Vaccines induce protective mechanisms in the body by mediating humoral and cellular immunity, achieving preventive effects. However, the cost of vaccination is relatively high, and the protection time for immunization is also relatively long. Of course, relevant experiments have also confirmed that vaccination can effectively reduce the degree of ileal lesions and pathogen shedding in infected piglets, thereby preventing porcine ileitis [15].

It is well known that the appropriate addition of antibiotics to feed can effectively control *LI* infection [16]. In recent years, antibiotic restrictions have been strengthened gradually, and vaccination has been shown to prevent PI infection in pigs [15,17]. Additionally, studies on the prevention or treatment through plant-derived extracts or probiotics have also been reported gradually [18,19]. As a functional compound fermentation product of various probiotics and enzymes, compound probiotics preparation can improve the growth performance and intestinal function of animals [20,21]. Our previous study also found that probiotic ferment [FAM] can improve growth performance, gut barrier, and function in piglets. Therefore, we aimed to reveal the mechanism that FAM alleviates negative performance in *LI*-challenged piglets by characterizing the changes in intestinal integrity and function and gut microbiota following FAM supplementation. Moreover, we provide a scientific basis for exploring the ability and application of FAM to alleviate *LI* infection.

## 2. Materials and Methods

The FAM used in this study was supplied by King Techina Feed Co., Ltd. from Zhejiang, China. The main ingredients are *Lactobacillus*, *Bacillus,* and their fermented products. Two main types of bacteria have a viable bacterial count of ≥1 × 10^9^ CFU/g. The additive amount of FAM in this experiment is 0.1% of the base diet.

### 2.1. Experimental Design and Treatments

**Figure 1:**
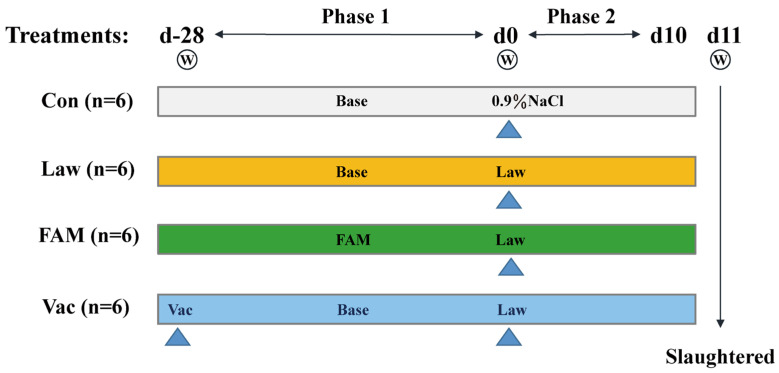
The major processes of experimental design and treatments. Base, base diet; FAM, base diet with FAM supplementation; Vac, piglets were vaccinated with commercial vaccine; Law, *LI* inoculation; NaCl, NaCl gavage treatment; W, weight.

As shown in Figure 1, there were four treatments and two phases in this experiment. Twenty-four 32 d old age Duroc × Landrace × Yorkshire hybrid healthy barrows (average weight about 9.1 kg, weaning at 21 d-old age) were randomly allotted to four treatments with six piglets in each pen. On the day of −28, six piglets in a pen (Vac) were vaccinated with 2 mL commercial vaccine (Enterisol^®^ Ileitis, Boehringer Ingelheim, Ingelheim am Rhein, Germany, B3903) according to the manufacturer’s instructions. Piglets in a group (FAM) were fed a basal diet supplemented with FAM, and the rest were fed a base diet only. Four weeks later, pigs in one pen (Con) were given 6 mL saline, and 18 other pigs were inoculated with 6 mL *LI* (Enterisol^®^ Ileitis, Boehringer Ingelheim, Ingelheim am Rhein, Germany, B3903). The order of inoculation was FAM group first, followed by Vac pigs, and finally, the remaining group (Law) of pigs. After that, the diarrhea rate, mental state, and feeding situation of each pig were observed and recorded over the next 10 days.

Each pen of approximately 10 m^2^ was equipped with a nipple waterer and a self-feeder, which had a safe distance from each other. Certainly, keepers should change their shoe covers and experimental clothes to clean themselves before approaching different pens. Additionally, the basal diet was formulated to meet the nutrient requirements of the National Research Council [22]. The composition and nutrient levels of the basal diet are presented in Table A1.

### 2.2. Growth Performance, Gross Pathology, and Sample Collection

Feed and individual body weight were recorded at d 28, d 0, and d 11 after 12 h of fasting (freely drinking) to calculate the average daily gain (ADG), average daily feed intake (ADFI), gain to feed ratio (G/F), and diarrhea incidence during the experimental period. The calculation of diarrhea incidence was as follows: Diarrhea incidence = the total number of diarrheal piglets/(number of piglets × number of days tested) × 100%.

After 24 piglets were slaughtered at d 11, the presence and extent of macroscopic lesions in each group were examined and scored under the judgment of unknown treatment. Specifically, the extent of ileal mucosa (thickening and hyperemia), mesentery (hyperemia), and mesenteric lymph nodes (swollen) were subjectively assessed using a score ranging from 0 (normal), 1 (mild), 2 (moderate), to 3 (severe).

Serum samples were collected by centrifugation at 3000 rpm for 10 min and stored at −80 °C for analysis (Thermo Fisher Scientific, Waltham, MA, USA). Ileal and colonic tissue samples about 0.1 × 0.3 cm^2^ were flushed with ice-cold saline, immersed in 2.5% glutaraldehyde fixative solution, and stored at 4 °C for electron microscope observation. Similarly, about 1 × 1 cm^2^ tissue samples of jejunum, ileum, colon, and mesenteric lymph node were immersed in 4% paraformaldehyde fixative solution (Servicebio, Wuhan, China) for light microscope observation. The fresh contents of the duodenum, ileum, colon, and pancreas were sampled into sterile tubes separately. Simultaneously, mucosa of jejunum and ileum were scraped by a sterile glass microscope slide at 4 °C. Then, all samples were rapidly frozen in liquid nitrogen and stored at −80 °C until analysis.

### 2.3. Digestive Enzyme Activities and Permeability

Approximately 0.2 g of duodenal contents, pancreas, and jejunal mucosa were taken to obtain a 10% tissue homogenate, respectively. The supernatant of duodenal contents and pancreas were taken to determine trypsin, lipase, and amylase activities, while the jejunal mucosa supernatant was taken to determine lactase, maltase, and sucrase activity in piglets. Additionally, intestinal permeability was detected through the contents of D-lactate, diamine oxidase (DAO), and endotoxin in serum.

All kits were purchased from the Nanjing Jiancheng Bioengineering Institute (Jiangsu, China), and the homogenate preparation and experimental procedures were carried out following the instructions.

### 2.4. Microscopic Pathology and Intestinal Morphology

Jejunum, ileum, colon, and mesenteric lymph node samples were placed in the dehydration box and into the dehydration machine for gradient alcohol dehydration, with 75% alcohol for 4 h, 85% alcohol for 2 h, 90% alcohol for 2 h, 95% alcohol for 1 h, anhydrous ethanol I and II for 30 min, benzene, xylene Ⅰ and xylene II for 5 min, and embedded in paraffin wax after dehydration. Additionally, the sections were further stained with hematoxylin and eosin (H and E) for morphological and pathological analysis. Finally, digital images would be obtained under a light microscope (Nikon, Tokyo, Japan).

Transmission electron microscopy (TEM, HITACHI, Tokyo, Japan) visualization of the ileum and colonic tissue and scanning electron microscope (SEM) visualization of the ileum tissue were conducted according to a previous study [23].

### 2.5. Periodic Acid Schiff Staining and Immunohistochemistry

Sections of paraffin-embedded ileum were dewaxed and rehydrated, immersed in 0.5% periodic acid for 10 min, and rinsed in distilled water. Then treated with Schiff’s reagent for 30 min in the dark and extensively rinsed in running tap water, stained with hematoxylin for 30 s, and rinsed with tap water again. Finally, dehydrated and sealed with neutral gum. Five regions were observed using an Eclipse Ci-L photographic microscope (Nikon, Tokyo, Japan) and analyzed by Image-Pro Plus 6.0 (Media Cybernetics, Rockville, MD, USA) after being sealed.

Observation of the distribution of Mucin 2 (Muc2) in the ileal tissue was performed by immunofluorescence. In brief, incubates slide with primary antibody (Servicebio, GB11344) overnight at 4 °C after three steps of being deparaffinized and rehydrated (the slices were placed in xylene I and II for 15 min, anhydrous ethanol I and II for 5 min, 85% ethanol for 5 min, 75% ethanol for 5 min, and washed with distilled water sequentially), antigen retrieval and serum blocking. The slides were washed three times; then, objective tissue was covered with a secondary antibody (Servicebio, GB21303, diluted with a concentration of 1:300) and incubated at room temperature for 50 min in dark conditions. Finally, sections were stained for 10 min using a 4′,6-diamidino-2-phenylindole (DAPI) solution (Servicebio, G1012). Images were obtained using ortho-fluorescent microscopy (Nikon, Nikon Eclipse C1) and an imaging system (Nikon, Nikon DS-U3).

### 2.6. RNA Chromogenic In Situ Hybridization

RNA in situ hybridization was used to determine the presence of *LI* in the ileum. According to the characteristics of the tissue, the protease K (ServiceBio G1234) was added to cover objectives and incubated at 37 °C for 20 min after the dewaxing and dehydration step. The pre-hybridization solution was added to incubate at 37 °C and removed 1 h’s later, followed by the addition of the *LI* 16S rDNA probe hybridization solution (5′-CY3-AACCGGAGCAGTCTCTCTAG-CY3-3′) containing CY3 label, which should be incubated in a humidity chamber and hybridized overnight at 42 °C. Finally, sections were incubated with DAPI (Servicebio, G1012) for eight minutes in the dark and then mounted. Images were observed under a microscope (Nikon, Nikon Eclipse C1) and collected by an imaging system (Nikon, Nikon DS-U3).

### 2.7. Measurement of Gene Expression by Real-Time Quantitative PCR (qRT-PCR)

To measure relative mRNA expression, the total RNA was extracted from samples according to the precise instructions of the Trizol method. The purity and concentration of total RNA were detected by a NanoDrop-1000 micronucleic acid analyzer. With RNA as a template, cDNA was synthesized using PrimeScript™ RT Master Mix (Takara, Tokyo, Japan). Next, the reaction mixture consisted of (20 μL): 10 μL SYBR Green Master Mix, 0.4 μL upstream and 0.4 μL downstream primers, 0.4 μL Rox Reference Dye II, 2.0 μL template DNA, and 6.8 μL ddH2O. qRT-PCR was executed on a LightCycler 480 System (Roche, Germany) using TB Green Premix Ex Taq™ (Takara, Tokyo, Japan). The relative expression of each gene was statistically analyzed based on the 2^−ΔΔCt^ method. The qRT-PCR primers and conditions are listed in Table A2.

### 2.8. Western Blot

The relative protein expressions in the colon were determined by western blot according to the previous study [24]. The total protein of the sample was extracted by RIPA lysis buffer. The protein concentration was determined by a BCA Assay Kit (Thermo Fisher Scientific, MA, USA). Five microlitres of 4× loading buffer were added to 15 μL of total protein and mixed well. This mixture was then used in a PCR instrument that incubated the mixture at 99 °C for 10 min. The denatured product was stored at −20 °C for later use. After electrophoresis separation, the denatured samples were transferred to polyvinylidene difluoride (PVDF) membranes. Next, the membrane was put into a 5% nonfat milk solution prepared with Tris-buffered saline and Tween-20 (TBST). The membrane was sealed on a shaker for two hours and then rinsed with TBST for one minute, hybridizing with primary and secondary antibodies. Then, the membrane was exposed to the Supersignal West Pico chemiluminescence substrate (Thermo Fisher Scientific, Waltham, MA, USA). The grayscale value of the protein bands was analyzed by template sequence Quantity One-4.6.2 software. The primary antibodies used in our study included an anti-ASBT antibody (diluted at 1:1000, Thermo Fisher PA5-18990), anti-CAT1 antibody (diluted at 1:800, Proteintech 14195-1-AP), anti-SGLT1 antibody (diluted at 1: 500, Abcam ab14686), anti-TCN2 antibody (diluted at 1: 200, LSBio LS-C373504), and anti-β-actin antibody (Abcam ab68477). The secondary antibodies were Goat anti-Mouse IgG (H+L) (diluted at 1:5000, Thermo Pierce 31431) and Goat anti-Rabbit IgG (H+L) (diluted at 1:5000, Thermo Pierce 31210).

### 2.9. Ileal and Colonic Luminal Microbiome Analysis

Microbial DNA was extracted from ileal and colonic contents using the TIANamp Stool DNA Kit according to the manufacturer’s instructions. DNA was amplified from the variable region 3 + 4 (V3+V4) of the bacterial 16S rRNA gene, with a primer sequence of (341F: 5′- CCTACGGGNGGWGCAG-3′, 805R: 5′- GACTACHVGGGTATCTAATCC-3′). PCR products were mixed in equal density ratios and purified with a GeneJet Gel Extraction Kit (Thermo Fisher Scientific). After the sequencing library was formed, the qualified library was sequenced by Illumina HiSeq 2500. The information analysis employs a Qiime (v1.91) standardized process and advanced open reference algorithms to assign sequences with ≥97% similarity to the same operational taxonomic unit (OTU).

Alpha diversity (Chao1, Observed-species, Shannon and Simpson) was calculated to reflect the bacterial diversity and richness. Beta diversity was determined using principal coordinate analysis (PCoA) and non-metric multidimensional scaling (NMDS). The relative abundance of microbiota was examined at different taxonomic levels, and linear discriminant analysis (LDA) effect size (LEfSe) analysis was performed to show the differences between the dominant species in different groups. Bubble plots, Spearman network plots, and scatter plots were used for correlation analysis and phenotype prediction. PICRUSt (phylogenetic investigation of communities by reconstruction of unobserved states) was used to generate predictive functional profiling of the intestinal microbiota.

### 2.10. Statistical Analysis

Data presented in the article are shown as means±SD, and *p* < 0.05 indicates a significant difference. The Gaussian distribution of data was analyzed by the Kolmogorov–Smirnov test (SPSS 26.0), and the variance of the data was analyzed by the homogeneity of variance test (SPSS 26.0). Statistical analysis was performed with one-way ANOVA followed by LSD’s multiple comparison tests to test the significance of differences.

## 3. Results

### 3.1. Piglets Infected with LI Showed Lower Growth Performance and Typical Pathological Symptoms

**Figure 2:**
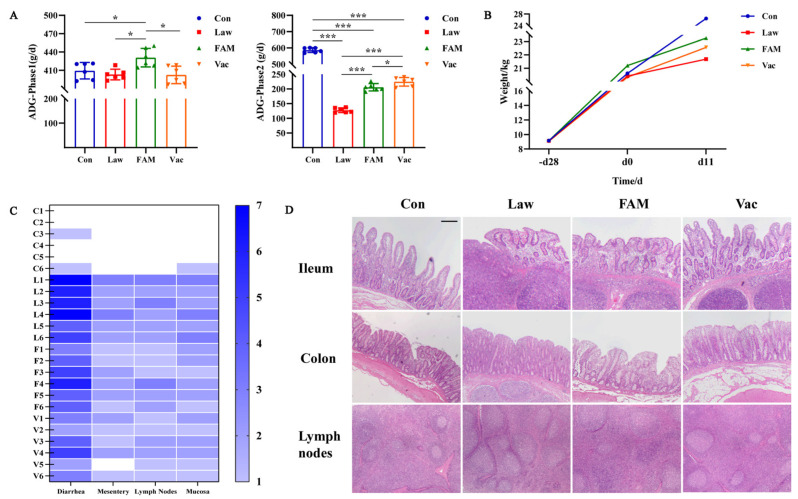
Piglets infected with *LI* showed lower growth performance and typical pathological symptoms. (**A**) The average daily gain (ADG) of piglets in phase 1 (left) and phase 2 (right). (**B**) Changes in piglet body weight throughout the experiment. (**C**) Pathological degree of diarrhea rate, mesenteric hyperemia, swelling of mesenteric lymph nodes, and ileal mucosal thickening. (**D**) Representative H and E-stained images of ileum, colon, and lymph nodes (top, scale bars = 200 µm). (*** *p* < 0.001, * *p* < 0.05).

The change of ADG during the experiment was divided into two phases (Figure 2A). In the first phase, piglets fed the FAM diet had greater ADG than the other groups (*p* < 0.05). While in phase 2, the ADG of the three groups inoculated with *LI* was significantly lower than in the Con group (*p* < 0.001) and higher in the FAM and Vac groups (*p* < 0.001). Table A3 showed the positive effects of FAM on ADFI and G/F in piglets before and after *LI* infection. Piglets in the Con group also had the highest final body weight throughout the experiment (Figure 2B). In terms of the pathological degree of diarrhea incidence (Table A3), ileal mucosa, mesentery, and mesenteric lymph nodes, the Law group was more serious than FAM and Vac groups. Moreover, the Vac group was slightly better than the FAM group (Figure 2C).

Some tissues showed typical pathological symptoms after *LI* infection (Figure 2D). Obvious Peyer’s patches in the ileal mucosa of *LI*-inoculated piglets were observed by H and E staining, which suggested an increased inflammatory response. We also found Peyer’s patches hyperplasia in both the ileum and colon in the Law group, while the colons of the other two groups only had edema phenomenon compared with that of the Con group. At the same time, the mesenteric lymph nodes of piglets infected with *LI* in the Law group are denser and more swollen in morphology. Similarly, the Law group was more severe than the FAM and Vac groups.

### 3.2. Intestinal Morphological Damage Induced by LI Could be Repaired by FAM

*LI* infection had a greater impact on the ileum and colon but less on the jejunum (Table 1). In the jejunum, we could observe that the ratio of villus height to crypt depth was reduced only in the Law group (*p* < 0.05). However, there were significant differences between the Law and Con groups in all parameters of the ileum and colon (*p* < 0.001). Simultaneously, FAM and vaccine had the same effect in reducing the increase in crypt depth caused by *LI* (*p* < 0.05). Furthermore, they also had the ability to reduce the ratio of villus height to crypt depth (*p* < 0.001), but the vaccine had a better effect than FAM (*p* < 0.05).

**Figure 3:**
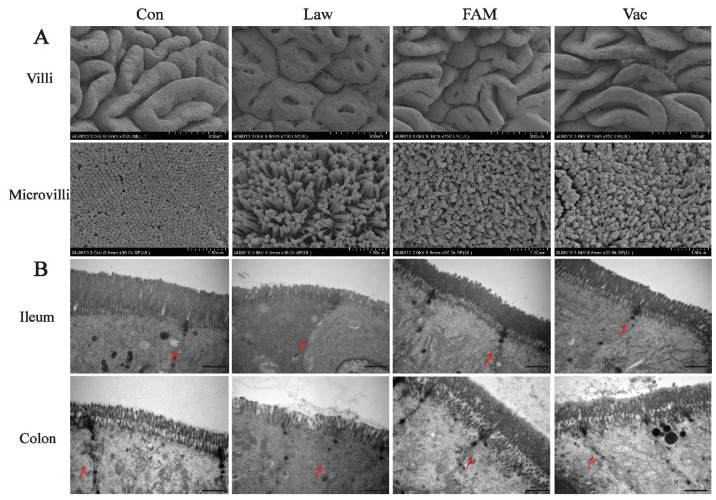
Ileal and colonic morphological damage could be repaired by FAM and vaccine. (**A**) Representative scanning electron microscope (SEM) images of ileal villi (bottom, scale bars = 300 µm) and ileal microvilli (bottom, scale bars = 1µm). (**B**) Representative transmission electron microscopy (TEM) images of ileal and colon microvilli (bottom, scale bars = 1µm, red arrows indicate epithelial cell junctions).

Based on the above results, we further observed the morphology of the ileum and colon by electron microscopy (Figure 3). Images of the ileal SEM showed the arrangement of villi and microvilli in each group, which were sparser and dirtier in the piglets infected with *LI* (Figure 3A). TEM images of the ileum and colon also showed that the microvilli in the Con group were tighter and denser, and the Con group had more complete intercellular junctions than the other groups (Figure 3B). Additionally, both FAM and vaccines could alleviate the above-mentioned negative effects caused by *LI*.

### 3.3. FAM Promoted Nutrient Utilization by Enhancing Digestive Enzyme Activity and Transporter Expression

**Figure 4:**
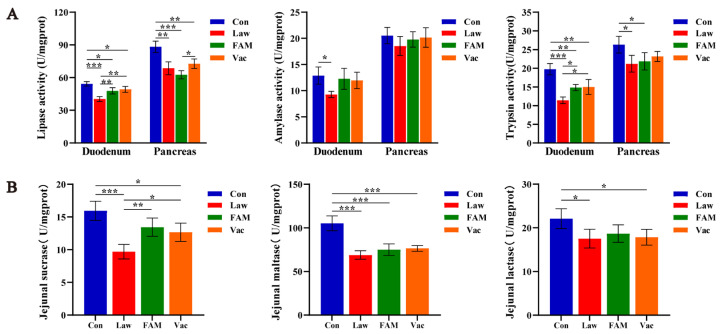
Effects of different treatments on the activity of digestive enzymes in piglets. (**A**) The activity of duodenal and pancreatic digestive enzymes (lipase, amylase, trypsin) among groups. (**B**) The jejunal maltase, lactase, and sucrase activity in each group. (*** *p* < 0.001, ** *p* < 0.01, * *p* < 0.05).

To explore the digestion of nutrients in piglets infected with *LI,* we first determined the activities of digestive enzymes, including amylase, trypsin, and lipase, in the duodenum and pancreas (Figure 4A). The results showed that, except for the pancreatic amylase activity, had no obvious change; the other indicators in the Con group were significantly higher than those in the Law group (*p* < 0.05). FAM and vaccine could also improve duodenal lipase and trypsin activities in *LI*-infected piglets (*p* < 0.05). Then, we examined the activity of jejunal disaccharidases (Figure 4B). It was learned that *LI* infection had a greater impact on the activities of maltase and sucrase (*p* < 0.001). Additionally, FAM and vaccine only increased sucrase activity but had no effect on lactase and maltase activity.

**Figure 5:**
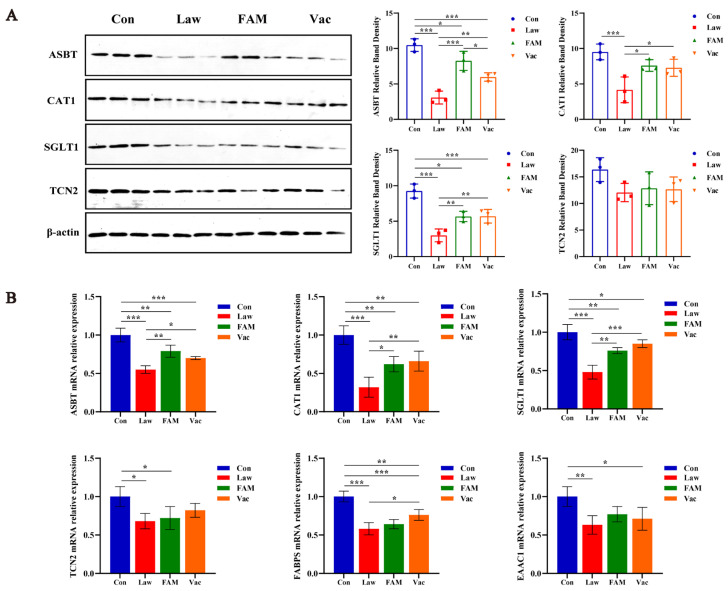
FAM and vaccine promoted the expression of ileal transporters. (**A**) Relative protein expression of the ileal apical sodium-dependent bile acid transporter (ASBT), cationic amino acid transporter (CAT1), sodium–glucose cotransporter 1 (SGLT1), and transcobalamin II (TCN2) among groups. (**B**) ASBT, CAT1, SGLT1, TCN2, fatty acid-binding protein (FABP), and excitatory amino acid carrier 1 (EAAC1) mRNA relative expression of ileum in each group. (*** *p* < 0.001, ** *p* < 0.01, * *p* < 0.05).

Additionally, the expression of some nutrient transporters in the ileum was also detected (Figure 5). Bile acid and vitamin B_12_ were the nutrients absorbed in the terminal ileum primarily. Figure 5A showed that FAM could significantly increase the expression of apical sodium-dependent bile acid transporter (ASBT) in *LI*-infected piglets at the protein level (*p* < 0.001), which is better than the vaccine (*p* < 0.05). However, there was no obvious improvement effect on transcobalamin II (TCN2). Furthermore, both of them contributed equally to improving cationic amino acid transporter (CAT1) expression and sodium–glucose cotransporter 1 (SGLT1) expression (*p* < 0.05). The above results were also verified from the detection at the mRNA level (Figure 5B). Additionally, we examined the fatty acid-binding protein (FABP) and excitatory amino acid carrier 1 (EAAC1) mRNA relative expression of ileum in each group, which found that only a vaccine could improve FABP expression in piglets infected with *LI* (*p* < 0.05) (Figure 5B). Certainly, the Con group had always maintained the highest expression at the protein and mRNA levels.

### 3.4. Reduction of LI Colonization Ameliorated Abnormal Differentiation and Function of Intestinal Epithelial Cells and Alleviated Severe Inflammatory Response in Piglets

**Figure 6:**
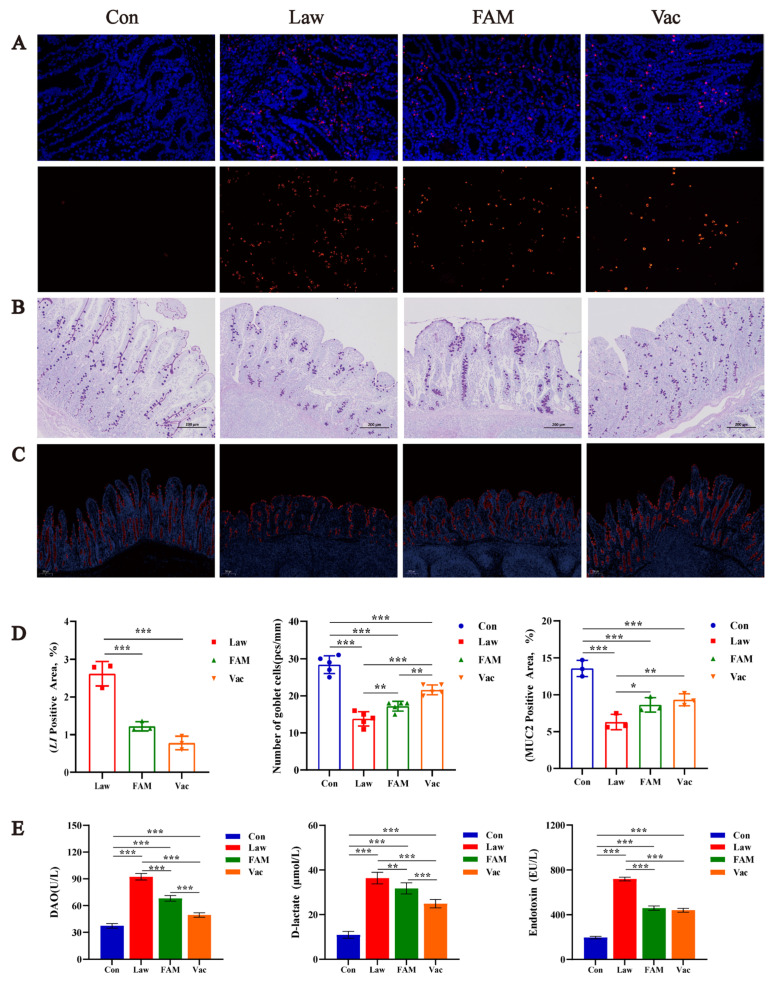
Reduction of *LI* colonization improved intestinal permeability and epithelial cell function. (**A**) Representative images of RNA in situ hybridization of piglet ileum colonized with *LI*. Nuclei were revealed with 4′,6-diamidino-2-phenylindole (DAPI) (blue), and positive expression was expressed by cy3 (red) to show the representative combined images (top) and individual *LI* images (bottom). (**B**) Representative images of PAS stained for piglet ileal goblet cells (scale bars = 200 µm). (**C**) Representative images of immunohistochemical detection of MUC2 (the imaging mechanism was the same as RNA in situ hybridization above, scale bars = 200 µm). (**D**) Quantification of *LI*, goblet cells, and MUC2 in the ileum. (**E**) Concentrations of D-lactate, diamine oxidase (DAO), and endotoxin in serum intestinal permeability indicators of piglets. (*** *p* < 0.001, ** *p* < 0.01, * *p* < 0.05).

RNA in situ hybridization was used to determine whether different treatments in each group would affect the colonization of *LI* in the ileum (Figure 6A). We observed that the ileal tissue of piglets infected with *LI* had different degrees of colonization, and there was nothing in the Con group. Both FAM and vaccine could reduce the risk of *LI* invading ileal tissue. Moreover, the number of ileal goblet cells and Muc2 were inversely correlated with the *LI* colonization amount described above (Figure 6B,C). As we counted (Figure 6D), the number of goblet cells in the ileum was significantly increased in FAM and vaccine groups (*p* < 0.01) but still less than in the Con group (*p* < 0.001). This series of negative changes might be responsible for the altered intestinal permeability (Figure 6E). It was *LI* infection that significantly increased intestinal permeability in healthy piglets (*p* < 0.001), and FAM and vaccine treatment could provide relief (*p* < 0.01).

**Figure 7:**
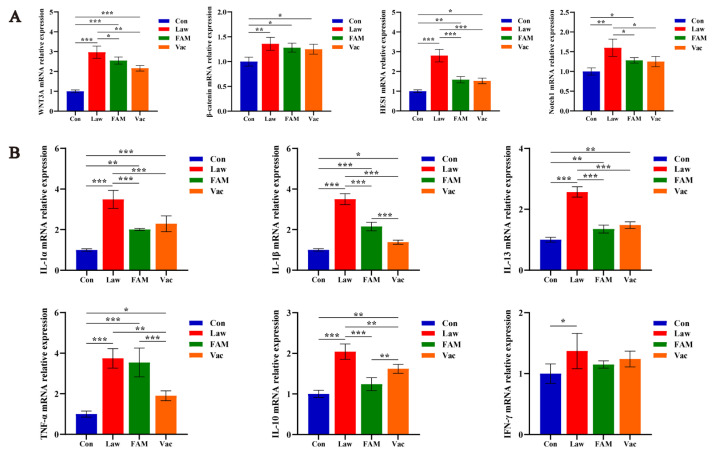
FAM and vaccine reduced severe inflammatory response in piglet ileum. (**A**) Relative mRNA expression of WNT3A, HES1, β-catenin, and Notch1 in piglet ileum among groups. (**B**) Relative mRNA expression of typical inflammatory cytokines (IL-1α, IL-1β, IL-13, TNF-α, IFN-γ, and IL-10) in the ileum of piglets. (*** *p* < 0.001, ** *p* < 0.01, * *p* < 0.05).

To further explore how *LI* reduced the number of goblet cells, we examined the mRNA relative expression of genes associated with enterocyte renewal and differentiation (Figure 7A). The results showed that both β-catenin/Wnt and Notch signaling pathways could be affected by *LI*, and the expression folds of WNT3A and HES1 in the Law group were more than two times that of the Con group. (*p* < 0.001). Except for β-catenin, the abnormality expression of genes in the Law group was significantly decreased in the FAM and Vac groups (*p* < 0.05).

The mRNA expression of ileal cytokine revealed the extent of the inflammatory response (Figure 7B). Results showed that *LI* could significantly increase the expression of inflammatory factors, which included all pro-inflammatory factors and one anti-inflammatory factor named IL-10 (*p* < 0.05). Simultaneously, both FAM and vaccine could effectively recover the genes other than IFN-γ and TNF-α to a lower expression (*p* < 0.05).

### 3.5. Structural and Functional Changes in the Ileal Microbiota of Infected Piglets Following FAM Supplementation

**Figure 8:**
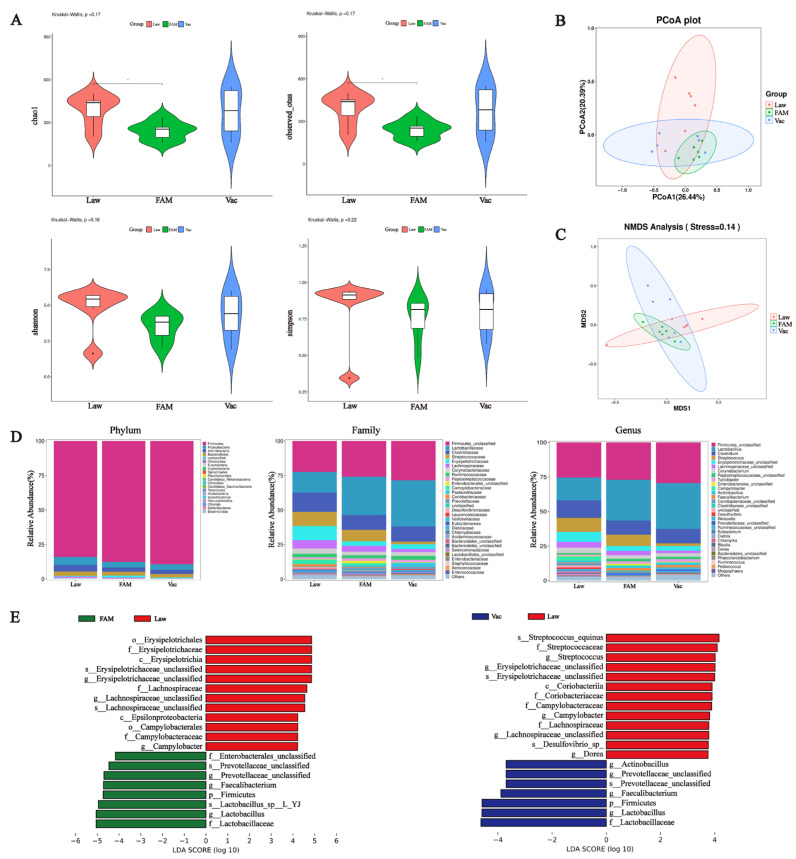
Changes in ileal microbiota structure in infected piglets. (**A**) α-diversity index including Observed-otus, Chao1, Simpson and Shannon. (B) Scatter plots obtained from principal coordinate analysis (PCoA) based on the Bray–Curtis phylogenetic distance metric. (**C**) Bacterial community non-metric multidimensional scaling (NMDS) analysis separated groups from each other. (**D**) Relative abundance of the microbial community at the phylum (left), family (middle), and genus (right) levels, respectively. (**E**) Histogram plots of enriched taxa based on linear discriminant analysis effect size (LEfSe) analysis revealed significant differences in the microbial community between groups. The left shows the results of the Law and FAM groups, while the right shows the Law and Vac groups. Bacterial taxa with linear discriminant analysis (LDA) score >3.5 were selected as biomarker taxa (*p*: phylum level, c: class level, o: order level, f: family level, g: genus level, s: species level). (*** *p* < 0.001, ** *p* < 0.01, * *p* < 0.05).

The α-diversity indices at four taxonomic levels were calculated to examine differences in gut microbiome richness and diversity across groups (Figure 8A). Interestingly, the Law group had significantly the highest richness indices of Chao1 and Observed-otus compared to the other groups (*p* < 0.05), while there was no difference in the diversity indices Shannon and Simpson. The PCoA plot and NMDS analysis also showed that the samples represented by points were closer between FAM and Vac groups, which implied a higher similarity between them (Figure 8B,C).

The structure of the intestinal microbiome at three levels and the LDA score were displayed to further verify the changes among the three groups. At the phylum level, Firmicutes was the most abundant phylum in all groups, followed by Proteobacteria and Actinobacteria. *Lactobacillaceae* and *Lactobacillus* in Firmicutes were also reduced at both the family and genus levels in the Law group (Figure 8D). LEfSe analysis indicated that FAM and vaccine increased the relative abundance of *Lactobacillaceae, Faecalibacterium,* and *Prevotellaceae* but decreased the relative abundance of *Erysipelotrichaceae* and *Campylobacter* (Figure 8E). Additionally, it was worth mentioning that *Desulfovibrio* abundance in the Law group was significantly higher than that in the Vac group.

**Figure 9:**
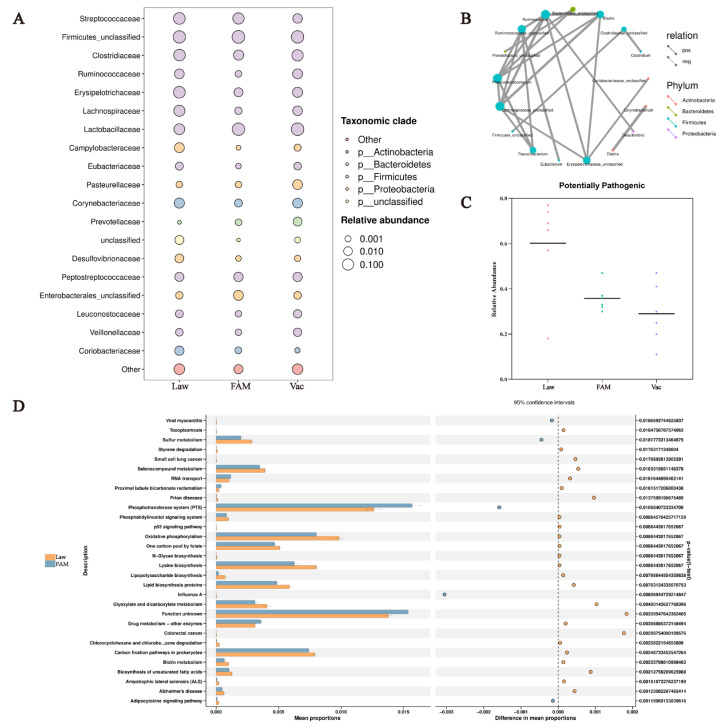
Prediction of ileal microbiota function in *LI*-infected piglets with different treatments. (**A**) Bubble plots at the family level showed the 20 most abundant microbiota by bubble size and color changes. (**B**) Spearman network plot of dominant attribute correlations at different phylum levels (default correlation coefficient |rho| > 0.8). (**C**) The relative abundance of samples in potentially pathogenic phenotypes was revealed by scatter plot. (**D**) The STAMP difference analysis plot showed predicted results of KEGG level 3 based on the PICRUSt2 function (with 95% confidence intervals).

As a differential microbe detected in the Proteobacteria phylum, *Desulfovibrio* has a positive relation with *Ruminococcaceae* (Figure 9B). The bubble plot at the family level also showed that the Law group had the highest abundance of *Desulfovibrio* and *Ruminococcus*, accompanied by an increased abundance of *Erysipelotrichaceae* and *Clostridiaceae* (Figure 9A). Therefore, we speculated that the above-mentioned changes lead to an increase in potential pathogens predicted by the bacterial phenotype (Figure 9C). The statistically significant difference (95% confidence interval) revealed by STAMP analysis was initial speculation for the effect of FAM supplementation on microbiota function in infected piglets (Figure 9D). The results showed that FAM promoted the function of RNA transport, proximal tubule bicarbonate reclamation, phosphotransferase system (PTS), and drug metabolism-other enzymes and reduced the increase in sulfur metabolism, lipopolysaccharide biosynthesis, and colorectal cancer caused by *LI* infection.

### 3.6. FAM Altered Diversity and Structure of Gut Microbiota in the Colon of Infected Piglets

**Figure 10:**
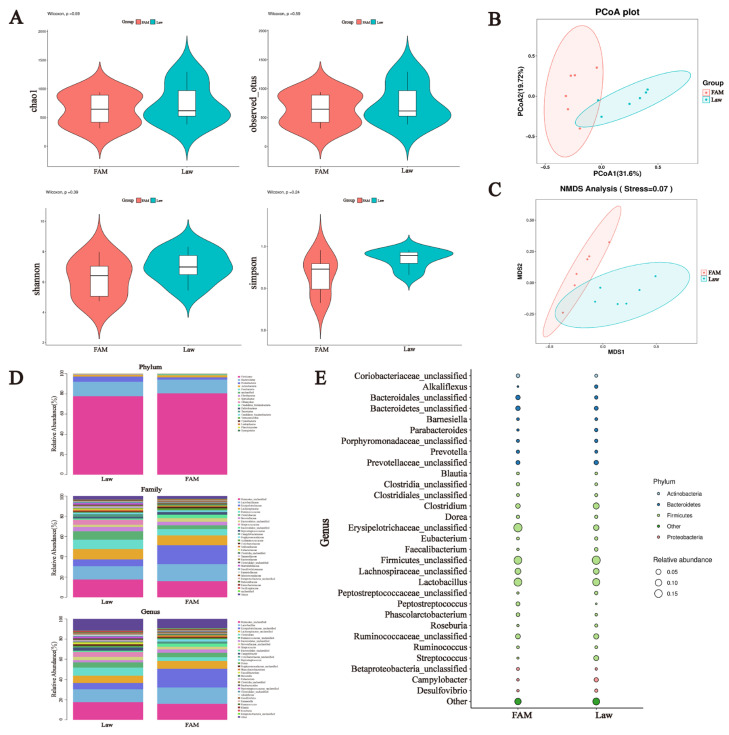
FAM altered the diversity and structure of gut microbiota in the colon of infected piglets. (**A**) α-diversity index including Observed-otus, Chao1, Simpson and Shannon. (**B**) Scatter plots obtained from PCoA based on the Bray–Curtis phylogenetic distance metric. (**C**) Bacterial community NMDS analysis separated groups from each other. (**D**) Relative abundance of the microbial community at the phylum (top), family (middle), and genus (bottom) levels, respectively. (**E**) The bubble plot at the genus level showed the 30 most abundant microbiota by bubble size and color changes.

Similar to the changes in the ileal microbiota, the α-diversity indicators between the two groups showed no significant difference but an upward trend in the Law group (Figure 10A). Observations from PCoA plots and NMDS analysis also found that samples within groups had no significant difference and could be distinguished well (Figure 10B,C). For a more detailed study of the structure of the microbiota, we analyzed the main microbiota at three levels again (Figure 10D). The phylum-level results showed FAM increased Firmicutes abundance and decreased Proteobacteria abundance. Moreover, the Law group had a higher abundance of *Clotrisdiaceae* and a lower abundance of *Lactobacillaceae* at the family and genus levels. In addition, *Desulfovibrio* was also detected as a differential genus between the two groups, which may be a manifestation of *LI* colonization in the gut of infected piglets (Figure 10E).

## 4. Discussion

Porcine ileitis caused by *LI* is a contagious intestinal disease that has a great negative effect on the growth and health of infected pigs. In this study, we revealed the mechanism that FAM alleviated negative performance in piglets by characterizing the impact of *LI* infection on intestinal integrity and function.

PI is endemic in many countries and causes severe economic losses in swine production systems worldwide due to the reduction of daily weight gain and feed conversion ratio [25]. In our study, piglets infected with *LI* all gained slower weight and had typical pathological symptoms. However, FAM still had a growth-promoting effect on infected piglets, especially in the first stage. This was consistent with our previous study, where the two main microbiota *Lactobacillus acidophilus* and *Bacillus subtilis* of FAM, improved ADG in weaned piglets significantly [26]. While other results of compound probiotics also promoted growth performance in piglets [27]. Therefore, as a green and safe feed additive, FAM not only has the foundation to alleviate piglet ileitis but also promotes growth in healthy piglets. This can be seen from the results of the first stage of the experiment. It was known that the jejunum and colon may be affected by *LI*, but the ileum is the main site of infection with severe pathology [2]. We speculated that this is the reason for the obvious pathological symptoms in the ileum and the fewer morphological changes in the jejunum. It was also worth mentioning that *LI* had a more negative effect on the colon than the jejunum in this experiment. Studies have reported that *LI* and *Mycoplasma hyopneumoniae* dual-challenged pigs had decreased ADG but normal ileal morphology [28], which was different from the results of increased ileal crypt depth and damaged villi in our study. While another study showed that weaned piglets naturally infected with *LI* had obvious subclinical symptoms in ileal histomorphology and could be regulated by a phytogenic additive [29]. Additionally, FAM established the foundation for alleviating the typical clinical symptoms and impaired intestinal morphology induced by *LI*.

Intact gut morphology is an important basis and indicator to reflect the efficiency of nutrient absorption [30]. Digestive enzymes in the gut are mainly secreted by pancreatic and small intestinal epithelial cells [31]. Referenced to negative changes in growth performance and intestinal morphology in infected piglets, we first speculated that piglets in each treatment had different digestive enzyme activities and verified the results later. In fact, it has been shown that *LI*-challenged pigs had lower ileal sucrase activity [30]. Helm et al. also found that *LI*-infected enterocytes lack sucrase-isomaltase, contributing to reduced pig digestive capacity [32]. Bile acids and vitamin B_12_ were nutrients absorbed primarily in the ileum, which played an important role in maintaining metabolic homeostasis and regulation [33,34]. Therefore, we further explored the effects of FAM and vaccines on the expression of ileal nutrient transporters. The results showed that the expression of transporter proteins was generally reduced in piglets infected with *LI*; both FAM and vaccine had a positive effect on ASBT but not on TCN2. A previous study has also found that the mRNA abundance of ileal nutrient transporters was reduced in *LI*-positive pigs [35]. The transport of glucose, amino acids, and fatty acids in the gut determines the ability of the body to metabolize proteins and fats [36,37]. Smith et al. reported that the ileum of infected piglets had lower expression of solute carriers, which were membrane proteins responsible for transporting nutrients and electrolytes [38]. Despite piglets infected with *LI* having a poor expression of CAT1, SGLT1, and FABP, significant improvements in FAM and Vac groups were still observed. It is well known that reduced nutrient absorption may lead to worse growth performance and higher diarrhea in piglets [39,40], so we believed that the improvement in intestinal morphology and nutrient absorption was the key to FAM alleviating the poor performance in infected piglets.

We have mentioned that the ileum is the main site of *LI* infection, so we tested its colonization by the method of RNA in situ hybridization. Certainly, previous studies have also explored the colonization of *LI* in different ways. Immunohistochemical images showed that lower antigen expression was observed in the ileum of challenged piglets fed a phytogenic feed additive [41]. Opriessnig et al. also reported that *Bacillus pumilus* probiotic feed supplements reduced the shedding of *LI* in the rectal swabs using the qPCR method [42]. Our previous research has shown that FAM enhanced the mucosal barrier by modulating gut microbiota-derived short-chain fatty acids and improved the structure and function of gut microbiota [26], which may affect the colonization of *LI* by regulating the number of beneficial and harmful microbiota. Among them, the two main microbiota *Lactobacillus acidophilus* and *Bacillus subtilis* of FAM, may play an important role. There was no doubt that the colonization of harmful bacteria in the gut would induce a series of negative effects. Studies reported that *LI* led to intestinal hyperplasia by stimulating cell division and altering cell integrity after invading the immature intestinal crypts [7,8]. Then, we predicted that the number and function of mature enterocytes would be inevitably affected. Indeed, results revealed that FAM and vaccine alleviated severe reduction in goblet cell and Muc2 numbers. Mucins are secreted by goblet cells to protect epithelial cells and are the first line of defense against pathogens [43,44]. Therefore, the reduction of *LI* colonization mediated by FAM and vaccine in our study was inseparable from the enhanced ability of goblet cells to secrete Muc2. Another report also revealed that *LI* infection of intestinal crypt cells was associated with specific depletion of secreted Muc2 in goblet cells [45]. Moreover, failure of mucosal epithelial cells maturation may lead to downregulation of nutrient transport genes and malabsorptive diarrhea [34]. Additionally, enhanced intestinal permeability and poor tight junctions between intestinal epithelial cells are indicative of intestinal mucosal damage [46,47]. In this study, both FAM and vaccine had a positive effect on the above indicators, which was the basis for our proposal that FAM alleviated absorption dysfunction and intestinal mucosal barrier damage. It is worth mentioning that the vaccine needs to be administered separately to piglets, and any antibiotic drugs are prohibited for two weeks before and after vaccination. Immunity also develops three to four weeks after vaccination, while FAM can be supplemented at the feed end, which is more convenient in comparison.

Usually, stem cells in intestinal crypts proliferate and differentiate into a variety of functional epithelial cells to form a constantly renewed epithelium [48]. However, the number and secretory capacity of goblet cells in *LI*-infected piglets were reduced in our study, so we supposed that aberrant expression of related pathways affected their differentiation. Studies have shown that Wnt/β-catenin and Notch signaling is the main regulatory mechanism for the proliferation and differentiation of intestinal stem cells [49]. The Notch signaling pathway mainly regulates the differentiation fate of secretory and absorptive intestinal epithelial cells, and inhibition of the pathway will increase the number of goblet and endocrine cells [50,51,52], while overexpression of Wnt/β-catenin signaling leads to the hyperproliferation of intestinal stem cells to develop intestinal proliferative crypts [53,54]. This is why we inferred that the improvement in goblet cell number and ileal absorptive capacity in the FAM group was associated with reduced overexpression of Wnt/β-catenin and Notch signaling pathways, despite researchers observing that *LI* infection was accompanied by a decrease in WNT3A transcript abundance [11]. Helm et al. also reported that the hyperplasia induced by *LI* might be partially driven by the heightened activity of both β-catenin/Wnt and Notch signaling pathways [31]. The infection mechanism of *LI* explored the immune response and showed staged differences with the change of infection time. The mucosal immune response may be in a suppressed state to facilitate the invasion and proliferation in the early stage of infection, a severe inflammatory response triggered during the peak phase and showed a trend of immune protection in the later stage [55]. Studies reported that serum TNFα was the first elevated cytokine due to *LI* infection, followed by the gradual increase in the secretion of IFN-γ and IL-6, while the serum concentrations of IL-10, IL-4, and TGFβ decreased [56,57]. In our study, FAM and vaccine down-regulated the severe inflammatory response induced by *LI*, even though there was still a certain gap from healthy piglets. Interestingly, IL-10 also increased with pro-inflammatory factors; we considered that increased secretion of anti-inflammatory cytokines might play a protective role by controlling inflammation and promoting recovery. Moreover, Nogueira et al. also found similar changes in IL-10 [57]. Regardless, the immune system will gradually return to normal levels and eventually resolve the digestive disturbance and barrier damage induced by *LI*.

The gut microbiota interacts with the intestinal mucosal barrier by regulating epigenetic modifications in the gut, thereby interfering with host immunity and metabolism [58,59]. Typically, an increased index of α-diversity reflects samples with higher richness and evenness [60]. However, the α-diversity of the ileal and colonic microbiota in the Law group showed an increasing trend, especially the Chao1 and Observed-otus indices of the ileum. Chen et al. also reported that major sub-OTUs of *Desulfovibrio* positively correlated with the diversity of gut microbiota [61], so we speculated that this is the reason for the increased diversity of the microbiota in the experiments. Firmicutes, Bacteroidetes, Proteobacteria, and Actinobacteria, are the four dominant phyla in the intestinal microbiota [62]. Although the proportions of the four dominant phyla differed between the ileum and colon, both FAM and vaccine increased Firmicutes abundance and decreased Proteobacteria abundance. Firmicutes produce short-chain fatty acids, suppress inflammation, and provide energy to intestinal epithelial cells to maintain gut health [63]. Both Lactobacillaceae and *Faecalibacterium* are beneficial bacteria of Firmicutes. Studies have shown that human CD4+/CD8α+ regulatory T cells induced by *Faecalibacterium prausnitzii* protect against intestinal inflammation [64], adhesion, and aggregation properties of *Lactobacillaceae* strains as protection against enteropathogenic bacteria [65]. At the same time, Proteobacteria include *Escherichia coli*, *Salmonella*, *Desulfovibrio*, *Helicobacter pylori,* and many other pathogenic bacteria [66]. *Erysipelotrichaceae* and *Campylobacter* have also been reported to be associated with host metabolic disorders and inflammatory diseases [67,68]. Therefore, FAM improved the ratio of beneficial and harmful bacteria to modulate the changes in the microbiota structure caused by *LI* infection.

As a taxon of *LI* at the genus level, *Desulfovibrio* was detected in the ileum and colon as the differential bacteria of Proteobacteria, which had a positive relation with Ruminococcaceae. A recent study showed that *Ruminococcus gnavu* lacking capsular polysaccharide could drive the inflammatory responses that characterize active inflammatory bowel disease [69]. This result was consistent with the colonization of *LI* in the ileum and may be one of the reasons for the increased potential pathogenicity in the Law group. *Desulfovibrio* is a typical bacterium that reduces sulfate to H_2_S under anaerobic conditions [70], while sulfide production may contribute to intestinal inflammation [71,72]. Therefore, it was not surprising that increases in *LI* colonization and *Desulfovibrio* were accompanied by an enhanced sulfur metabolism. Lipopolysaccharides modulated intestinal epithelial permeability and inflammation in a species-specific manner [73]; this inflammation was characterized by enhanced inflammatory cytokine production and epithelial damage [74]. In contrast, PTS is a widely distributed and highly efficient carbohydrate transport system found in most bacterial species that catalyzes the simultaneous phosphorylation and import of cognate carbohydrates, which is the key to the regulation of cell physiology by the gut microbiota [75]. We speculated that FAM alleviated *LI*-induced intestinal damage by altering these metabolic pathways in this study. However, the mechanism that *LI* induces changes in gut microbiota and probiotics stabilize intestinal homeostasis to be further revealed and confirmed in future studies to precisely demonstrate the essential role of probiotics in the therapeutic effect of *LI*- infection.

## 5. Conclusions

In conclusion, probiotic ferment can reduce the colonization of *Lawsonia intracellularis* in the ileum, improve intestinal damage, barrier function, and microbiota structure, and enhance digestive enzyme activity and nutrient transport proteins expression, thereby improving piglet growth performance, which has the effect of preventing ileitis in pigs. Moreover, it will provide a scientific basis for the prevention of pig ileitis and its application in the aquaculture industry.

## Figures and Tables

**Table 1:** Intestinal morphology of jejunum, ileum, and colon.

Item	Con	Law	FAM	Vac	*p* Value
Jejunum					
Villus height (μm)	523.4 ± 21.6	505.5 ± 22.5	518.4 ± 18.1	513.3 ± 20.4	0.349
Crypt depth (μm)	254.5 ± 11.0	278.3 ± 11.4	270.5 ± 12.0	262.4 ± 10.4	0.350
Villus height/Crypt depth	2.08 ± 0.11 ^a^	1.82 ± 0.09 ^b^	1.92 ± 0.09 ^a,b^	1.96 ± 0.07 ^a,b^	0.099
Ileum					
Villus height (μm)	473.3 ± 18.2 ^a^	407.3 ± 15.6 ^c^	427.2 ± 14.7 ^b,c^	454.1 ± 20.7 ^a,b^	0.007
Crypt depth (μm)	212.1 ± 9.7 ^c^	297.0 ± 14.0 ^a^	269.1 ± 13.3 ^b^	265.4 ± 12.4 ^b^	0.000
Villus height/Crypt depth	2.23 ± 0.13 ^a^	1.37 ± 0.03 ^d^	1.59 ± 0.02 ^c^	1.71 ± 0.08 ^b^	0.000
Colon					
Crypt depth (μm)	480.3 ± 16.1 ^c^	597.3 ± 20.9 ^a^	553.4 ± 18.3 ^b^	559.4 ± 19.5 ^b^	0.000

^a–d^ Different superscripts within a row indicate a significant difference (*p* < 0.05).

## Data Availability

The data of raw sequences derived from microbiome analysis are available in the Sequence Read Archive database at NCBI under accession number PRJNA842217, accessed on 27 December 2020. https://dataview.ncbi.nlm.nih.gov/object/PRJNA842217?reviewer=thu79q6ida498hpip48r5dq2p6.

## Appendix A

**Table A1:** Composition of the basal diet (DM basis).

Ingredients	Content, %	Nutrient Composition ^2^	Content
Corn	63	Digestible energy, kcal·kg^−1^	3500
Soybean meal	18	Crude protein, %	18.8
Puffed soybeans	8	Calcium, %	0.75
Fish meal	4	Available phosphorus, %	0.4
Whey powder	3	Lysine, %	1.4
Dicalcium phosphate	1	Threonine, %	0.87
Limestone	0.77	Methionine, %	0.42
Salt	0.4	Tryptophan, %	0.21
L-Lysine	0.5		
Threonine	0.2		
DL-methionine	0.1		
Tryptophan	0.03		
Premix ^1^	1		
Total	100		

^1^ vitamin A, 9000 IU; vitamin B_1_, 3 mg; vitamin B_2_, 6.5 mg; vitamin B_6_, 3 mg; vitamin B_12_, 20μg; vitamin D_3_, 3000 IU; vitamin E, 30 IU; vitamin K_3_, 2.5 mg; biotin, 0.1 mg; folic acid, 0.9 mg; pantothenic acid, 14 mg; niacin, 40 mg; choline, 400 mg; Fe, 100 mg; Cu, 100 mg; Mn, 30 mg; Zn, 80 mg; I, 0.2 mg; Se, 0.3 mg. ^2^ The contents in nutrient composition were calculated values.

## Appendix B

**Table A2:** Target genes detected in the study and their primers sequences.

Gene	Forward Primer (5′-3′)	Reverse Primer (5′-3′)	GenBank Number
GAPDH	CCAAGGAGAACTCAAGATTTGGC	TGCCGTGGGTGGAATCATAC	XM_021091114.1
IL-1β	ATGCCAACGTGCAGTCTATG	CATGCAGAACACCACTTCTCT	NM_214055.1
TNF-α	GGCCCAAGGACTCAGATCAT	GGCATACCCACTCTGCCATT	NM_214022.1
IL-1α	AACCTGGATGAGGCAGTGAAA	AGCACTCACAAACAGTCGGG	NM_214029.1
IL-10	GAAGACGTAATGCCGAAGGC	AGGGCAGAAATTGATGACAGC	NM_214041.1
IL-13	TTGCTCTCACCTGCTTTGGT	GCATAGGGGTGTCTTCTGGT	XM_005661643.3
IFN-γ	GTTTTTCTGGCTCTTACTGC	CTTCCGCTTTCTTAGGTTAG	NM_213948.1
HES1	AACACGACACCGGATAAACCA	GGAATGCCTCGAGCTATCTTTT	NM_001195231.1
WNT3A	GCTCTCGGGGCGGACT	CAACAGCCAGGGACCACC	XM_003123621.4
Notch1	CCCGAGTGGACAGGTCAGT	GTCGTCGATGTTTTCGCTGC	XM_021081037.1
β-catenin	GGCTTGGAATGAGACTGCTG	GGGTCCATACCCAAGGCATC	XM_013981492.2
SGLT1	CGTCATCTACTTCGTGGTGGT	AGAAGCTCCAACCGGCCA	NM_001164021.1
TCN2	TGCCTGGACCTGCGAAATAG	TTGAGGCTGTGCAGGTAGTG	XM_021071885.1
ASBT	TGCCGCCCTCCCCACAAC	GGACTGACCCTGGACTCTGACC	NM_000452.3
CAT1	TCACGCTCATGATGCCCTAC	TGGAGCCTAAAAGGCTGGTG	NM_001012613.1
EAAC1	AACCCTTTCCGATTCGCCAT	GCTGTGGCGGTGATACTGAT	NM_001164649.1
FABPS	AGTTGACCATCACTACCGGG	AACTGAACCACTGTCTTGACC	NM_001004046.2

## Appendix C

**Table A3:** Growth performance and diarrhea incidence.

ITEM	Con	Law	FAM	Vac	*p* Value
d −28~d 0					
Initial BW (kg)	9.15 ± 0.44	9.12 ± 0.39	9.14 ± 0.47	9.06 ± 0.34	0.872
Final BW(kg)	20.61 ± 2.17	20.41 ± 2.63	21.20 ± 1.85	20.33 ± 2.58	0.534
ADG (g/d)	409.3 ± 13.5 ^b^	403.2 ± 8.7 ^b^	430.7 ± 15.2 ^a^	402.5 ± 14.4 ^b^	0.040
ADFI (g/d)	646.4	619.2	648.1	623.9	
F/G	1.58	1.54	1.5	1.55	
d 0~d 10					
Initial BW (kg)	20.61 ± 2.17	20.41 ± 2.63	21.2 ± 1.85	20.33 ± 2.58	0.477
Final BW (kg)	26.48 ± 2.33 ^a^	21.68 ± 3.39 ^b^	23.26 ± 2.94 ^b^	22.57 ± 2.85 ^b^	0.000
ADG (g/d)	586.6 ± 14.9 ^a^	127.5 ± 7.9 ^d^	205.7± 12.6 ^c^	224.4 ± 14.7 ^b^	0.000
ADFI (g/d)	1020.7	698.3	791.6	823.5	
F/G	1.74	5.48	3.85	3.67	
Diarrhea incidence, %	3.3	58.3	43.3	31.7	

^a–d^ Different superscripts within a row indicate a significant difference (*p* < 0.05).
